# The Influence of Nb on the Synthesis of WO_3_ Nanowires and the Effects on Hydrogen Sensing Performance

**DOI:** 10.3390/s19102332

**Published:** 2019-05-20

**Authors:** Dario Zappa

**Affiliations:** SENSOR Laboratory, Department of Information Engineering (DII), University of Brescia, Via Valotti 9, 25133 Brescia, Italy; dario.zappa@unibs.it; Tel.: +39-030-371-5767

**Keywords:** metal oxides, hydrogen sensing, nanowires, tungsten oxide, chemical sensors, niobium

## Abstract

Hydrogen sensing is becoming one of the hottest topics in the chemical sensing field, due to its wide number of applications and the dangerousness of hydrogen leakages. For this reason, research activities are focusing on the development of high-performance materials that can be easily integrated in sensing devices. In this work, we investigated the influence of Nb on the sensing performances of WO_3_ nanowires (NWs) synthetized by a low-cost thermal oxidation method. The morphology and the structure of these Nb-WO_3_ nanowires were investigated by field emission scanning electron microscope (FE-SEM), high-resolution transmission electron microscope (HR-TEM), X-ray diffraction (XRD), Raman and X-ray photoelectron (XPS) spectroscopies, confirming that the addition of Nb does not modify significantly the monoclinic crystal structure of WO_3_. Moreover, we integrated these NWs into chemical sensors, and we assessed their performances toward hydrogen and some common interfering compounds. Although the hydrogen sensing performances of WO_3_ nanowires were already excellent, thanks to the presence of Nb they have been further enhanced, reaching the outstanding value of more than 80,000 towards 500 ppm @ 200 °C. This opens the possibility of their integration in commercial equipment, like electronic noses and portable devices.

## 1. Introduction

Metal oxides (MOX) represent a vast class of materials of interest for various scientific communities, ranging from physics to chemistry, from material science to engineering [1,2,3] and recently in medicine for exhaled breath analysis [4,5,6]. The majority of metal oxides are in principle sensitive to gases. However, in order to be performing materials for the fabrication of chemical sensors, they need to fulfill specific requirements such as sensitivity, selectivity, stability, fast response and recovery times.

In the field of chemical sensing, metal oxides have been investigated in different forms, starting from thick and thin films [7,8,9,10,11]. In recent years, nanostructured metal oxides and in particular one-dimensional (1D) nanowires have attracted wide attention, especially for the fabrication of conductometric sensors [12,13,14,15]. These devices are among the most performing and cheapest devices that may be integrated, for example, into portable detection systems, due to their easy readout, small size and low cost.

One of the gas sensors’ applications that is becoming quite popular is hydrogen sensing [16,17,18,19], due to the increasing demand of industrial processes that require an accurate detection and monitoring [20,21]. For example, hydrogen is the main fuel of solid oxide fuel cells (SOFCs), which are considered the next generation of energy conversion devices. SOFCs have much higher efficiency compared to traditional combustion engines (more than 60%), and there is an aim to replace traditional combustion engines in almost every application [22,23,24,25]. However, hydrogen is extremely dangerous as it is tasteless, odorless, colorless and it cannot be detected by human senses. Its use requires accurate sensing, to avoid any potential leakage that could lead to harmful and dramatic consequences. In fact, hydrogen is highly flammable in air, with flammability limits that range from 4% to 75% in volume in nitrogen atmosphere. If mixed with oxygen, flammability limit reaches 94% [26].

All metal oxides have limitations regarding the selectivity between different chemical species, and they require high temperature operation [27]. To overcome MOX selectivity issues, different strategies have been proposed, including the doping of the material [28,29]. For the detection of hydrogen gas, pristine metal oxides have been explored already [16,30]. Among them, tungsten oxide has proved to be a good candidate thanks to its excellent performances [31,32,33,34,35,36,37]. Boudiba et al. reported in 2013 the promising hydrogen sensing performances of WO_3_ nanoparticles loaded with Pd catalyst (1%), prepared by chemical route [38]. In 2014 Esfandiar et al. incorporated Pd-WO_3_ ribbon-like nanostructures on graphene oxide (GO) and partially reduced graphene oxide [36]. Gasochromic devices were reported also, fabricated by sputtering a Pt layer onto the surface of WO_3_ films. Differently from chemosensors, in these devices the changes in UV-VIS spectra were evaluated by alternate exposures to H_2_ and air [34]. A huge increase of the sensing performances was achieved thanks to the fabrication of quasi-1D structures. Annanouch et al. reported in 2016 the synthesis and the sensing properties of Pd-decorated WO_3_ nanoneedles via a two-step chemical vapor deposition (CVD), exhibiting a response 750 times higher than pristine WO_3_ [31].

The doping of tungsten oxide by niobium has been studied rather extensively, but is now attracting a significant interest in many applications, in particular for energy storage [39], adsorption of radioactive waste ions [40] and catalysis [41,42]. However, niobium-doped tungsten trioxide was used rarely as active material for the fabrication of chemical sensors. In 2015, Kruefu et al. successfully synthetized Nb-loaded hexagonal WO_3_ nanorods by hydrothermal method, resulting in a response of 10 toward 500 ppm of SO_2_ [43]. Other works reported in literature, instead, used the hydrothermal technique to fabricate W-doped niobium oxide. Yu et al. investigated the NO_2_ gas sensing performance of tungsten doped niobium oxide nanorods, demonstrating ppb-level detection at room temperature [44]. The same research group also reported the exploitation of hydrothermal W-doped niobium oxide nanorods for hydrogen and methane sensors [45,46]. Despite the poor literature, thanks to their synergistic properties the combination of nanostructured niobium and tungsten oxides looks very promising for the selective detection of hydrogen gas.

The aim of this work is to report a facile technique for the synthesis of Nb-doped tungsten oxide nanowires, for the fabrication of superior hydrogen sensing devices. The thermal oxidation process does not require any precursor, it produces no waste reagents and it could be faster than the hydrothermal technique. Moreover, nanostructures are synthetized directly on the active transducers used for the fabrication of the sensors, avoiding the transfer processes and thus increasing both the yield and the stability of the devices. By combining the properties of niobium and tungsten oxides with the high surface-to-volume-ratio of nanowires, it is possible to enhance further the hydrogen sensing performance without affecting the morphology of the material. 

Thanks to the facile synthesis technique, mainly consisting in a controlled oxidation process in vacuum, and the outstanding sensing performances of these Nb-WO_3_ nanowires, the proposed method could be easily extended into real mass-scale production processes with no efforts. These nanowires are well suited to be integrated into arrays for portable sensing devices or electronic noses (e-noses), to further increase the selectivity of the instruments toward hydrogen [47,48,49,50].

## 2. Materials and Methods

### 2.1. Synthesis of the Nanostructures

Thermal oxidation technique was used to synthetize pristine WO_3_ and Nb-WO_3_ nanowires. For pristine samples, a metallic tungsten film was deposited on 2 × 2 mm^2^ alumina substrates (Kyocera, Japan, 99% purity) by RF magnetron sputtering (100 nm thickness, 100 W argon plasma, 100 °C, ≈0.55 Pa) starting from a pure W target ( >99.9% purity) [51]. For Nb-WO_3_, instead, we inserted a number of niobium stubs into the W target, depositing a metallic tungsten-niobium alloy on the substrates. Basically, three different batches of samples were prepared: pristine WO_3_, WO_3_ + Nb(4) and WO_3_ + Nb(12), according to the number of niobium stubs inserted.

The oxidation process was performed inside a custom vacuum tubular furnace. The pressure inside the furnace was set at 100 Pa. The influence of the gas flow was studied, and the effect of the oxidation temperature was evaluated in the range of 500 °C–700 °C. Prior to structural characterization, samples were annealed in air at 400 °C for 24 hours, to completely oxidize the residual tungsten layer.

### 2.2. Morphological and Structural Characterization

A field-emission scanning electron microscope (FE-SEM, model LEO 1525, ZEISS), coupled with an Oxford energy dispersive x-ray analysis (EDX), was used to investigate the morphology, the elemental composition and stoichiometry of fabricated nanostructures.

Microscopic morphological investigations were carried out using a JEOL 2100 analytical transmission electron microscope (TEM), with LaB_6_ Gun and emSIS Tengra CCD, operated at 200 kV in TEM mode.

X-ray diffraction spectroscopy (XRD) was performed using an Empyrean diffractometer (PANalytical, Almelo, The Netherlands), mounting a Cu-LFF (λ = 1.5406 Å) tube and operated at 40 kV – 40 mA. Spectra were obtained by Bragg-Bentano geometry, using a linear PIXcel 1D with a large-β nickel filter and were recorded in the 20°–80° range.

Raman spectra were measured by using a He-Cd laser (442 nm) connected to a HORIBA confocal optical microscope and monochromator (iHR320), with a grating of 1800 g/mm and a Peltier-cooled Synapse CCD. Spectra were recorded in the wavelength range 200–1000 cm^−1^.

Near ambient pressure X-ray photoelectron spectroscopy (NAP-XPS, SPECS GmbH, Germany, Al Kα monochromatized source) allows the chemical analysis of the surface of materials in the hundreds of Pa range atmosphere. In our test, the pressure inside the cell was 300 Pa, the atmosphere composition was 80% nitrogen and 20% oxygen to simulate in-operando working conditions and measurements were performed at room temperature. All reported binding energy (BE) data was calibrated using the C1s peak of the residual C contamination at the surface of the materials. For chemical elements quantification, relative sensitivity factors were provided by SPECS for Al Kα source (θ ≈ 54°).

### 2.3. Chemical Sensing Measurements

Three different batches of sensing devices were prepared at optimal conditions, with different concentration of Nb added: pristine WO_3_, WO_3_ + Nb(4) and WO_3_ + Nb(12). Interdigited contacts (IDEs) were deposited on the substrates by a two-steps deposition: (1) TiW adhesion layer by DC magnetron sputtering (100 nm, 70 W argon plasma, 300 °C, ≈0.55 Pa); (2) Pt electrodes, using the same parameters used for the adhesion layer (thickness ≈1 μm). A platinum heater was deposited on the backside of alumina substrates via the same two-steps process. Devices were finally mounted on packages using electro-soldered gold wires. To stabilize the sensing material and the electrodes/heater, sensors were aged at 400 °C for one week prior to the electrical measurements. 

Flow-through technique was used to investigate the conductometric response of fabricated sensors [52]. Devices were placed in a stainless-steel chamber of 1 dm^3^ volume, inside a climatic chamber (Angelantoni, Italy, model MTC 120) set at 20 °C. The temperature of sensors was controlled independently by using Thurlbly-Thandar PL330DP power supplies.

Sensors were thermally stabilized at the desired working temperature for 8 hours prior to the effective measurement, in presence of a humid air flow (Relative Humidity - RH = 50% @ 20 °C) of 200 sccm. Test gases with a certified composition and concentration, supplied by SIAD SpA (Italy), were mixed with dry air by MKS Instrument mass flow controllers, maintaining a total flow of 200 sccm. After the 30 min exposure to a fixed concentration of target gas, synthetic air flow was restored for 60 min, to allow the recovery of the baseline. A fixed voltage of 1 V was applied to the sensors (Agilent E3631A power supply), measuring at the same time the conductance of each sensor using dedicated picoammetters (Keithley 486). The response is calculated by the variation of the conductance using the following formulas for reducing and oxidizing gases, respectively:

(1) $$Response=\frac{G_{Gas}-G_{Air}}{G_{Air}}$$

(2) $$Response=\frac{R_{Gas}-R_{Air}}{R_{Air}}=\frac{G_{Air}-G_{Gas}}{G_{Gas}}$$

where $R_{Gas}$ and $G_{Gas}$ are respectively the sensor resistance and conductance in presence of gas, and $R_{Air}$ and $G_{Air}$ in synthetic air. Sensing performances were evaluated towards various concentrations of hydrogen and some common interfering chemical compounds: ethanol, acetone, nitrogen dioxide and ammonia. Firstly, a temperature screening was performed, in the range of 100–500 °C, to identify the optimal working temperature for each chemical compound. Secondly, calibration curves were measured, at the optimal working temperature for hydrogen detection. Power-law trend line was calculated also, as it is very common for metal oxide conductometric devices [53]:

(3) $$Response=A*C^{B}$$

where *C* is the concentration of the target compound, *A* and *B* are constants related to the material composition and the involved surface chemical reactions. Moreover, detection limits, response and recovery times, and hydrogen-interfering gases’ response ratios were calculated.

## 3. Morphologic and Structural Investigations

**Figure 1:**
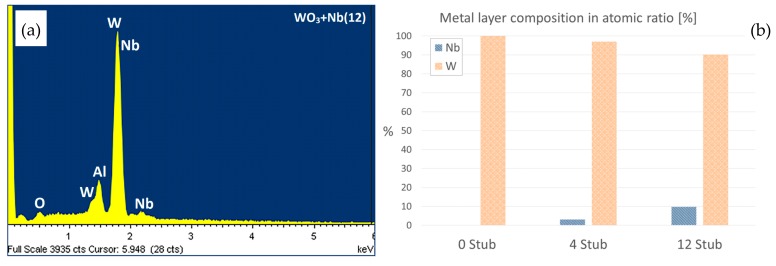
WO_3_ + Nb(12) EDX spectrum (**a**) and histogram of W-Nb atomic ratio (**b**) of films deposited with different Nb stub inserted in tungsten target.

Prior to the oxidation process, an initial assessment on the amount of niobium introduced inside the tungsten films was performed by EDX analysis. Figure 1a reports a typical WO_3_ + Nb(12) EDX spectrum obtained from sample, in which only W, Nb, Al and O lines are present. The last two lines are due to alumina (Al_2_O_3_) substrates being used for the characterization. Because the sputtered films are thin (≈100 nm), the interaction volume of the e-beam at 10 keV during EDX measurements excites the substrate also. The estimated relative percentage of niobium in the tungsten films using different Nb metal stubs is reported in Figure 1b. With only four stubs inserted, the presence of Nb was identified as 3% atomic. Increasing the number of stubs to 12, we detected about 9.5% of atomic niobium inside the tungsten matrix.

Afterwards, samples were moved inside the custom furnace and oxidized. Firstly, the influence of the atmosphere composition on the growth of the nanowires was evaluated. Keeping the same base pressure of 100 Pa, pristine WO_3_ samples were oxidized in the presence of three different gas flows that were injected: 10 sccm of Ar, 10 sccm of O_2_ and no flow. The gas flow was kept low enough to not affect the base pressure inside the furnace. The SEM pictures of three different samples are reported in Table 1. The oxidation temperature and time were kept at 550 °C and 1 h, respectively.

We observed that the use of a pure oxygen flow inside the furnace leads to a completely oxidized layer, that is yellow colored, grain-like and without any nanowires. In the absence of any gas flow, samples started to show some very small dots, which were the nucleation sites for the nanowires growth. The use of an argon flow, instead, resulted in a uniform mat of disordered nanowires completely covering the tungsten film. It seems that, in order to get the nanowires, the amount of available oxygen should be limited. If oxygen is abundant, the oxidation process is too fast, leading to the formation of a grain-like film. In oxygen-lacking atmospheres, instead, the oxidation process is slower, helping the nucleation of WO_3_ seeds on the tungsten film that allow the formation of nanowires. The growth process is similar to the one previously reported for the synthesis of CuO nanowires by thermal oxidation in air [54].

After the identification of the optimal atmosphere, a temperature screening in the range of 500–700 °C was performed, to determine the influence of the temperature on the oxidation process and to observe the effect of the presence of niobium in the nanowire synthesis. Results are reported in Table 2. The pressure was fixed at 100 Pa, using the optimal gas flow of 10 sccm of argon. Oxidation time was set at 1 h.

**Figure 2:**
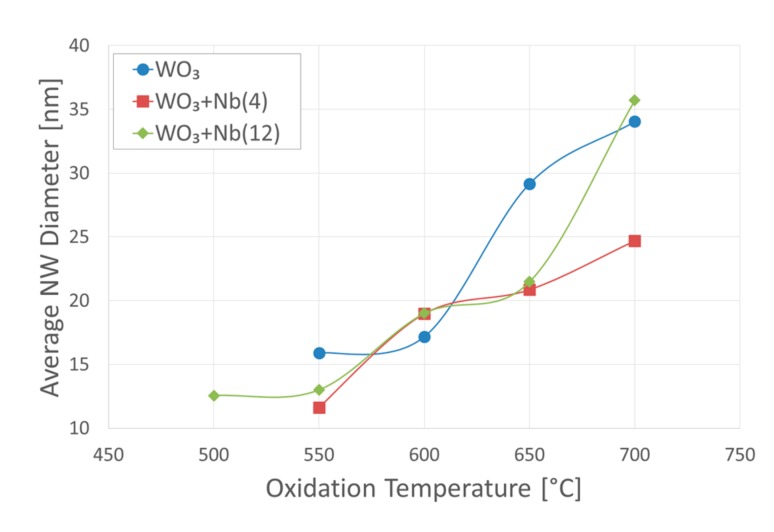
Trend of nanowires’ (NWs) diameters synthetized at different temperature, for pristine and Nb-WO_3_ materials.

The lowest oxidation temperature (500 °C) was not enough to promote the growth of nanowires, leading to the formation of few nucleation seeds on the surface of the material. The addition of niobium inside the tungsten film seems to promote slightly the nucleation: in the case of 12 Nb stubs, nanowires became clearly visible, even if they were very sparse and small. Starting from 550 °C, nanowires started to nucleate and grow on the substrates, increasing their dimensions according to the oxidation temperature. In Figure 2, we report the average diameter of the nanowires synthetized at various oxidation temperatures. Standard deviation is not represented in the graph for clarity reasons, but has been reported in Table S1. Through a statistical analysis, we can confirm that the size of the nanowires is normally distributed, with an average diameter that ranges from slightly more than 10 nm (550 °C) to about 30 nm (700 °C). Interestingly, the presence of niobium seems to promote the synthesis of nanowires, but does not increase their size.

**Figure 3:**
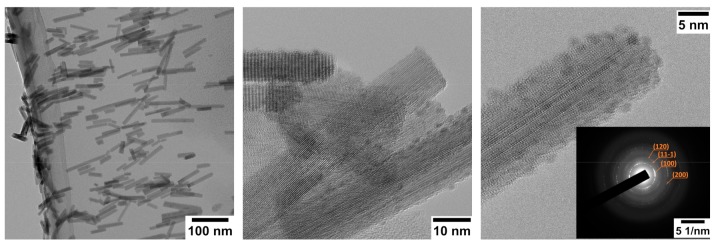
HR-TEM of WO_3_ + Nb(12) nanowires. Inset: SAED pattern, showing (200), (100), (11-1) and (120) diffraction rings.

Detailed morphological studies are required to understand the shape and the composition of single nanowires. For TEM observations, nanowires were dispersed on carbon grids, after being detached from the substrates via mechanical scratching. High-resolution TEM images confirm the crystallinity and the size of pristine and Nb-WO_3_ materials (Figure 3). The nanowire appears as a single crystal of monoclinic WO_3_, as confirmed by selected area electron diffraction (SAED) analysis [55]. We were not able to detect any secondary phases in the presence of niobium. However, the structure is strongly defected, as we can see from the high surface roughness. It is worth mentioning that the dot-like structures on the edges of nanowires are defects and irregularities of the surface, and not a secondary material as in the case of surface functionalization, for example. We can observe some structural defects on pristine WO_3_ also, but they are less extended. 

**Figure 4:**
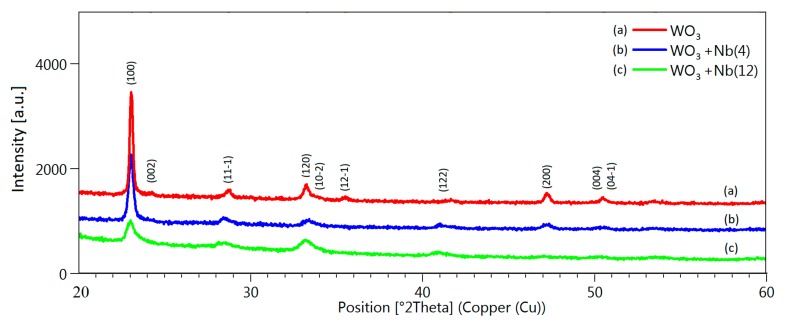
XRD spectra of pristine (**a**), WO_3_ + Nb(4) (**b**) and WO_3_ + Nb(12) (**c**) nanowires.

The crystalline structure was investigated also by x-ray diffraction (XRD), to confirm the results of SAED analysis. Pristine and Nb-WO_3_ spectra are shown in Figure 4. We were not able to individuate any significant peak at positions higher than 60°; therefore, we are reporting the data only in the 20°–60° range. Detected diffraction peaks can be attributable to the monoclinic crystalline structure of WO_3_ (*m*-WO_3_) for all three materials (JCPDS card number 01-083-0950). With the introduction of niobium in the lattice, there is a progressive reduction of the intensity and a broadening of the diffraction peaks, including the main peak at 23.1° (100). Moreover, we observe a small shift in the peaks toward lower angles. For example, the WO_3_ peak at 23.1° (100) and 28.7° (11-1) move to 23.0° and 28.4° in WO_3_ + Nb(12), respectively. Diffraction peaks at 35.5°, related to (12-1) plane, and 50.5°, related to (004) and (04-1) planes, almost disappear in Nb-WO_3_ samples. However, there are no new peaks attributable to niobium phases in these samples. From the information obtained by TEM and XRD, we can conclude that the crystalline structure of Nb-WO_3_ nanowires is still monoclinic as pristine WO_3_, but the presence of niobium introduces many defects in the structure that reduce the overall crystallinity and increase the surface roughness.

**Figure 5:**
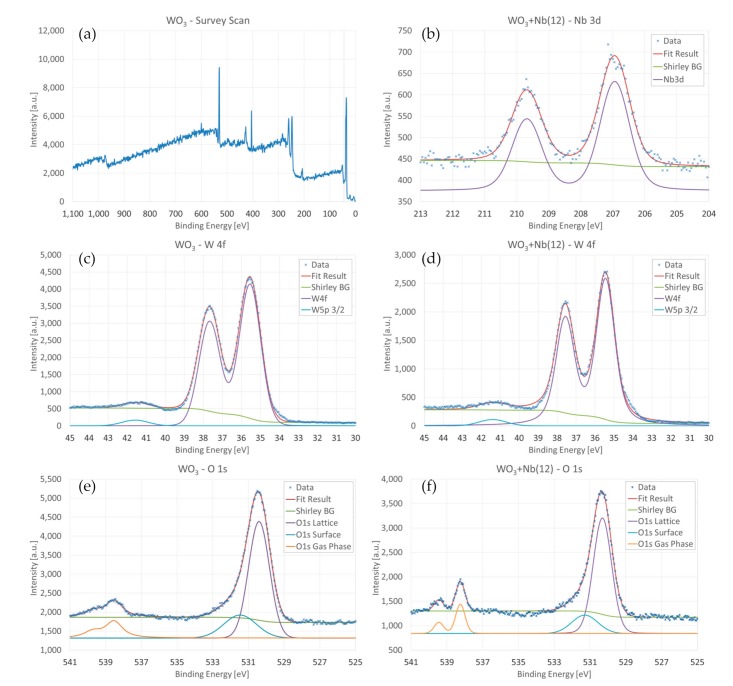
(**a**) XPS survey scan of pristine WO_3_. (**b**) XPS scan for Nb3d lines on WO_3_+Nb(12). (**c**,**d**) XPS investigations of W4f lines on pristine WO_3_ (**c**) and WO_3_+Nb (**d**) samples. (**e**,**f**) XPS investigations of O1s lines on pristine WO_3_ (**e**) and WO_3_+Nb(12) (**f**) samples.

XPS measurements were performed to assess the chemical composition of the surface of the materials. The survey scan performed on pristine WO_3_, reported in Figure 5a, only identifies spectral lines related to W, O, N and C atoms. For Nb-WO_3_ materials (Figure 5b), Nb lines were also detected. The W4f core-level binding energy (BE) displays a rather strong dependency on the oxidation state of the W atom [56,57,58,59]. The energies at 35.5 eV and 37.6 eV positions, with a peak separation of 2.1 eV, are related to W4f_7/2_ and W4f_5/2_ for tungsten atoms in W^6+^ oxidation state. There is no sign of other tungsten states (W^4+^ or W^2+^), and we did not record any significant shift between pristine and Nb-WO_3_ nanowires. The higher energy peak at 41.5 eV is assigned to W5p_3/2_, which always goes with W4f_7/2_. However, its position is nearly unchangeable and therefore less significant.

Regarding oxygen, we may observe an intense peak at 530.4 eV and a second component at 531.5 eV. The main peak is attributed to O^2-^ ions forming the crystalline lattice, whereas the secondary line could be related to weakly adsorbed species or subsurface O^-^ ions [60]. As for tungsten, the presence of niobium atoms does not modify significantly the position of the oxygen energy lines, but we observed a small broadening of both peaks, which could be due to the chemical bonding between Nb and O atoms in the structure. Because the XPS measurements were performed in-operando using a synthetic atmosphere (80% N_2_, 20% O_2_) at 300 Pa pressure, the doublet peaks at 538 eV and 539.7 eV are attributed to molecular O_2_ in gas phase [61,62].

Figure 5b shows the Nb3d spectrum recorded on WO_3_ + Nb(12) samples, exhibiting two peaks at 207.0 and 209.7 eV. The peaks represent the Nb3d_5/2_ and Nb3d_3/2_ components, respectively, with a spin-orbit splitting of 2.7 eV, which correspond to Nb^5+^ oxidation state (Nb_2_O_5_) [63,64]. However, we were not able to observe a separation between Nb_2_O_5_ and WO_3_ crystallites in SEM and TEM images, suggesting that the samples are bulk doped. Semiquantitative calculation has been performed to determine the relative concentration of Nb atoms in WO_3_ + Nb(12) samples [64]. From these calculations, relative [Nb]/[W] concentration resulted in 10.1%, in line with EDX investigations on metallic sputtered layers. The overall concentration of niobium in WO_3_ + Nb(12) samples is estimated as ≈3%.

**Figure 6:**
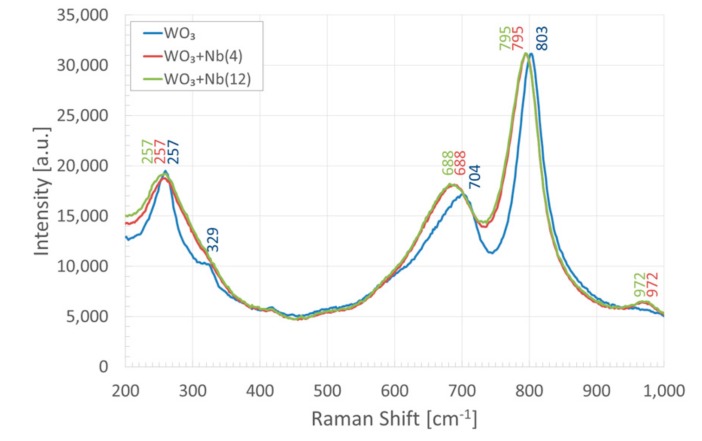
Raman spectra of pristine (**blue**) and Nb-WO_3_ (**red** and **green**).

WO_3_ is composed of a network of corner-shared WO_6_ octahedron units, which are connected to each other by W–O–W or hydrogen bonds through water bridges, with terminal W=O bonds at the surface of the clusters [51]. Raman spectra of pristine and Nb-WO_3_ nanowires are reported in Figure 6. In pristine sample, the bands at 257 and 329 cm^−1^ correspond to O−W−O bending modes of the bridging oxygen of monoclinic tungsten oxide (*m*-WO_3_), while the bands at 704 and 803 cm^−1^ are the corresponding stretching modes [65,66]. In the presence of niobium, the bending mode at 257 cm^−1^ widens, overlapping with the one at 329 cm^−1^. This could hint that the structure is more defective and less crystalline. At the same time, the two stretching modes shift to 688 and 795 cm^−1^, respectively.

Curiously, Nb_2_O_5_ shares the similar octahedral structure of WO_3_, which can be derived by the joining of blocks of corner-shared NbO_6_ octahedra with connected blocks sharing the edges of the octahedron. More specifically, the form most commonly encountered is monoclinic *H*-Nb_2_O_5_ which has a complex structure, with a unit cell containing 28 niobium atoms and 70 oxygen, where 27 of the niobium atoms are octahedrally coordinated and one is tetrahedrally coordinated [67]. Nevertheless, we did not detect any peak attributable to monoclinic or orthorhombic Nb_2_O_5_ phases in our measurements [68,69,70]. The shifts of the two stretching modes suggest that the bond distance increases and the binding energy decreases. Combining XPS with Raman results, we can speculate that niobium atoms randomly replace tungsten atoms inside the octahedral structure, effectively doping the nanowires. However, the amount of niobium introduced does not seem to influence significantly the distortion of the octahedral structure, as the Raman spectra of WO_3_ + Nb(4) and WO_3_ + Nb(12) are almost identical.

The band around 970 cm^−1^ is assigned to the stretching mode of the terminal W=O bond; this mode is usually related to the presence of water, as is common for all types of tungsten trioxide hydrates [71]. However, W=O stretching mode is shifted from the literature value (950 cm^−1^), and it is present only when niobium is introduced in the structure. It is possible that this stretching mode arises from the substitution of tungsten atoms with niobium in the octahedron units, introducing some defects in the crystal structure. 

## 4. Functional Characterizations as Chemical Sensor

**Figure 7:**
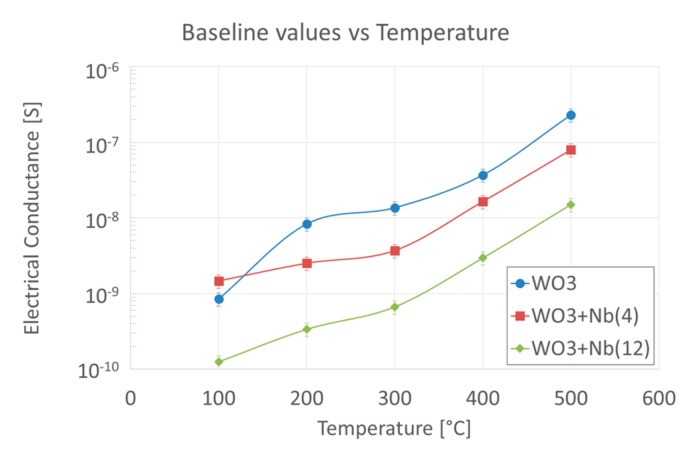
Baseline electrical conductance values of pristine and Nb-WO_3_ nanowires at RH = 50% @ 20 °C, in synthetic airflow, versus the operating temperature.

For the fabrication of the sensing devices, we have selected an oxidation temperature of 600 °C, which leads to the formation of a dense mat of nanowires with an average diameter lower than 20 nm. Fabricated sensor devices were characterized according to their electrical and functional properties. Firstly, the effects of the operating temperature and the presence of niobium on the electrical conductance baseline of the devices were investigated, under a pure synthetic airflow at RH = 50% @ 20 °C. Data are reported in Figure 7, which shows that for all three batches of samples the electrical conductance increases with the operating temperature, as expected for n-type metal oxides such as WO_3_. Interesting, the baseline of the devices decreases as we increase the amount of niobium inside the material. Nevertheless, the trend of the baseline dependence on temperature of Nb-WO_3_ samples is similar to the one of pristine WO_3_.

**Figure 8:**
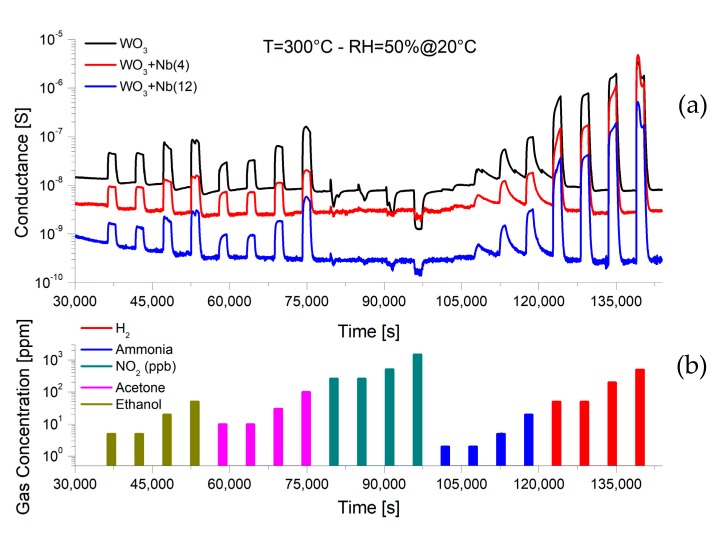
(**a**) Dynamic response of for pristine and Nb-WO_3_ nanowires, at 300 °C and RH = 50% @ 20 °C, for various concentrations of chemical compounds. (**b**) Gas injections and corresponding concentrations.

Sensor devices were exposed to various concentration pulses of target chemical compounds, while their electrical conductance was monitored by the measurement chamber. Figure 8 reports an example of the dynamic response of the fabricated three batches at 300 °C. In the presence of reducing gases, such as hydrogen, ethanol, acetone and ammonia, the electrical conductance of the devices increases. Contrarily, in the presence of an oxidizing gas such as nitrogen dioxide, the conductance of the devices decreases. As we said, this is the typical behavior of *n*-type metal oxide materials, which confirms that the macroscopic semiconducting nature of the material has not been influenced by the presence of niobium. Moreover, from Figure 8, it is possible to observe the lower baseline conductance of Nb-WO_3_ samples compared to the pristine one.

**Figure 9:**
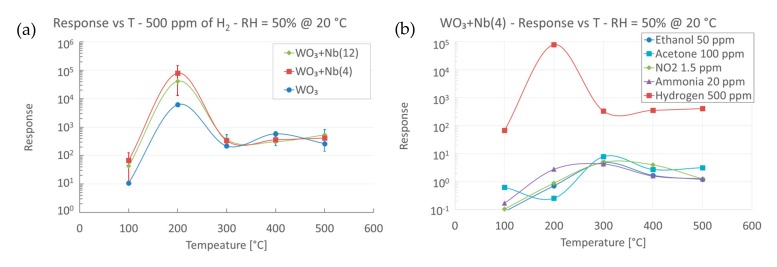
(**a**) Response versus operating temperature in the presence of 500 ppm of hydrogen, for pristine and Nb-WO_3_ nanowires (RH = 50% @ 20 °C). (**b**) Temperature screening of WO_3_ + Nb(4) nanowires in the presence of fixed concentrations of hydrogen, ethanol, acetone, nitrogen dioxide and ammonia (RH = 50% @ 20 °C).

The three batches of samples were characterized in the presence of hydrogen and other common interfering compounds, at various operating temperature and at a relative humidity fixed at 50% @ 20 °C. We decided to keep humidity constant during these preliminary investigations, at a value close to application requirements. Before mass production, however, the influence of humidity on the response of Nb-WO_3_ nanowires should be addressed, as we previously performed for pristine WO_3_ [51]. In Figure 9a, we compared the response of pristine and Nb-WO_3_ nanowires in the presence of 500 ppm of hydrogen, which is almost two order of magnitude smaller than the Lower Explosive Limit (LEL) of hydrogen (4%). Moreover, this value is in the range that has to be detected in many applications, including safety devices. Investigating the effect of the operating temperature on devices’ responses, we recorded a similar trend among the three different materials. All of them exhibit the optimal response at 200 °C, which settles to a lower value for temperatures higher than 300 °C. The introduction of niobium inside the WO_3_ nanowires considerably improves the response, both in case of WO_3_ + Nb(4) and WO_3_ + Nb(12) devices. In particular, best results are obtained by the smallest addition of niobium, which leads to the outstanding response of 8 × 10^4^ at 200 °C that is one order of magnitude higher than pristine WO_3_. Moreover, this low optimal operating temperature (200 °C) allows the use of these sensors in applications in which power consumption and dissipated heat are concerned.

Figure 9b reports the response to 500 ppm of hydrogen compared to other interfering compounds, for WO_3_ + Nb(4) devices. Error bars have not been reported for visual clarity, but they are estimated as <15% of the response. We may observe that the response toward hydrogen is higher than any other tested compounds, in their relevant concentration ranges. Therefore, we can confirm that these devices are extremely selective to hydrogen, potentially reducing the risk of false detection. It is worth mentioning that the response spectra of the pristine WO_3_ and WO_3_ + Nb(12) is similar to the one reported in Figure 9b for WO_3_ + Nb(4).

**Figure 10:**
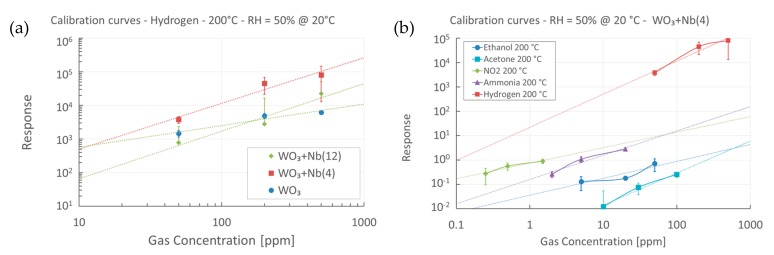
(**a**) Calibration curve towards hydrogen for pristine and Nb-WO_3_ nanowires, at 200 °C and RH = 50% @ 20 °C. (**b**) Calibration curve towards hydrogen and interfering compounds for WO_3_ + Nb(4) devices, at 200 °C and RH = 50% @ 20 °C.

Preliminary calibration curves for all target compounds were extracted from the data. Figure 10a reports the direct comparison of the hydrogen calibration curves for pristine and Nb-WO_3_ nanowires, whereas Figure 10b represents the curves of all target compounds for WO_3_ + Nb(4), at 200 °C and RH = 50% @ 20 °C. The extracted coefficients for all target compounds and materials are reported in Table S2. In particular, the *B* coefficient is usually related to the stoichiometry of the surface reactions [53]. From Table S2 we can speculate that ethanol and ammonia sensing reactions are not influenced strongly by the presence of niobium, as the slope of the curves remain constant. On the contrary, the sensing mechanism of acetone and nitrogen dioxide seems to be affected by its presence. Regarding hydrogen, we already observed a strong enhancement of the response, which could be related to a different sensing mechanism due to niobium. However, the amount of niobium introduced does not seem to affect this mechanism, as the slope of the curves for WO_3_ + Nb(4) and WO_3_ + Nb(12) is the same.

**Figure 11:**
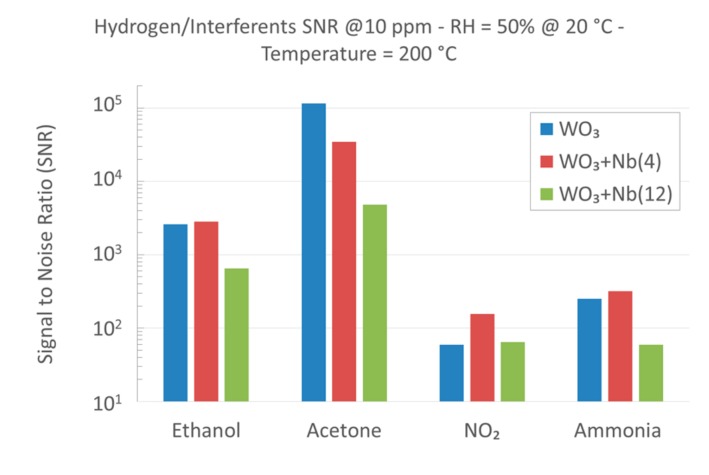
Signal to Noise Ratio (SNR) between 10 ppm of hydrogen and 10 ppm of interfering compounds, for pristine and Nb-WO_3_ nanowires, at 200 °C and RH = 50% @ 20 °C.

Selectivity among different chemical compounds is a common limitation of metal oxide materials when they are integrated into conductometric devices, and is usually very difficult to achieve [72,73]. As we inferred, Nb-WO_3_ materials are very promising for the detection of hydrogen, even in the presence of other interfering compounds. Signal to Noise Ratio (SNR) was defined as the ratio between the response towards 10 ppm of hydrogen and 10 ppm of interferents (Figure 11). If data samples were not available for a certain compound, response was extracted from the calibration curves reported in Table S2. SNR calculated for pristine WO_3_ nanowires is already excellent, in particular in the presence of volatile organic compounds (VOCs) like ethanol and acetone. A small addition of niobium improves the SNR of NO_2_ and NH_3_ even further, but at the maximum amount it decreases considerably. Nevertheless, WO_3_ + Nb(4) devices have proved to be very robust in the selectively detection of hydrogen gas.

**Figure 12:**
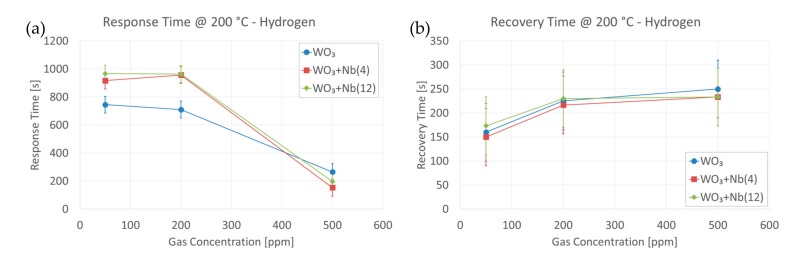
Response (**a**) and Recovery (**b**) times calculated from the dynamic responses of all samples, at 80% signal variation, in the presence of various concentrations of hydrogen. Operating temperature = 200 °C and RH = 50% @ 20 °C.

The response and recovery times of all samples were calculated, to evaluate the effect of Nb on the dynamic performances of the sensors. Figure 12a,b report the results obtained in the presence of different concentrations of hydrogen, averaged on different samples from the same batch. It is worth pointing out that the size of the test chamber and the gas flow used limit the dynamic performances of the devices. The volume of the test chamber is about 1 dm^3^ (1 L), and it takes about 4–5 min to fill completely with an injected gas flow of 200 sccm. Data were collected every 30 s due to the system specification. Response times show a slight advantage of pristine WO_3_ samples at low concentrations, while recovery times are almost identical and definitely limited by the test chamber.

During all measurements, the response stability was investigated by performing repeated measurements on at least four identical sensors under the same experimental conditions, that enabled to estimate a maximum uncertainty of 15%. The lifetime of the present sensors can be estimated to be >1 year, over which the samples are still working without any evident deterioration of the surface, exhibiting a small drift (<20%) which is typical of metal oxide materials [74].

The widely-adopted sensing mechanism of semiconductor gas sensors is associated with the change of the electrical conductance of the material, which depends on the gas molecules or ions to detect and chemisorbed oxygen species (O^−^, O^2−^ and O_2_^−^) on the surface of the semiconductor [53]. In *n*-type semiconductors, these adsorbed oxygen ions on the surface trap free electrons from the conducting band of the semiconductor, leading to the formation of a depletion region on the surface of the material. Because in nanostructures the size of this depletion region is comparable to the one of the material itself, they are almost depleted, which has dramatic effects on the overall electrical conductance. When the material is exposed to a reducing gas, such as ethanol, adsorbed oxygen ions interact with the gas molecules, releasing the trapped electrons that could return to the conduction band and therefore narrowing the depletion region. The opposite happens when an oxidizing gas is introduced on the surface [53,75]. For *p*-type materials, such as copper oxide, this behavior is completely specular [76]. The detailed sensing mechanism is reported in literature, including the role of humidity [36,37,38,77,78].

WO_3_, which is the most stable form of tungsten oxide, is composed mainly of W^6+^ states and is known to have catalytic properties, enhancing the adsorption rate of gaseous species such as hydrogen and ethanol. However, crystalline forms of WO_3_ can also exist with an oxidation state of 5+, especially if the structure is defective. This 5d0 configuration in W, may lead to the formation of numerous defect traps throughout the lattice, which contribute to its unique sensing properties. According to our XPS investigations, pristine and Nb-doped nanowires exhibit W^6+^ states mostly. Therefore, we can suppose that trap states in pristine WO_3_ play a minor role in the sensing process. On the other hand, the Nb atoms found on the surface of the Nb-WO_3_ nanowires have a 5+ oxidation state, leaving the Nb 4*d*-band void of electrons and resulting in the creation of desired defect traps [79]. These energy traps may contribute to the increase of the sensing performances of Nb-doped devices.

In the presence of hydrogen, we may have different phenomena occurring simultaneously on the surface of the material, which both result in an increase of the electrical conductivity. The first mechanism involves the Pt electrodes deposited on the sensing layer. Platinum is a well-known catalyst for the dissociation of hydrogen molecules. These gas molecules adsorb onto the Pt surface, and then can subsequently dissociate and diffuse through to the Pt/WO_3_ interface. Adsorbed oxygen ions interact with dissociated hydrogen forming water molecules and releasing electrons, as reported in Equation (4).

(4) $$2H_{ads}+\;O_{ads}^{-}\to H_{2}O+\;e^{-}$$

The second mechanism is related to the hydrogenation of tungsten oxide (H-WO3), which leads to the formation of hydrogen tungsten bronze [38,80]:

(5) $$xH_{ads}+\;WO_{3}\to H_{x}WO_{3}$$

Regarding the decrease of the electrical conductance baseline in Nb-WO_3_ samples, we may attribute this effect to two different reasons. The first one is that Nb-doping inside the WO_3_ crystalline lattice leads to the formation of local nano-heterojunctions, which enlarge the depletion regions on the surface of the nanowires. Oxygen ions may adsorb on defect states created by the presence of Nb atoms, further increasing the depletion region. The second hypothesis is that the increased surface roughness, due to defects in the crystalline structure, leads to an increased surface area of the sensing material. Therefore, there could be a higher number of oxygen ions adsorbed on the surface of the nanowires, which trap more free electrons from the conducting band of the semiconductor, further depleting the surface of Nb-WO_3_ and thus increasing the electrical resistance. The decrease in hydrogen response observed at operating temperature above 200 ° C may be interpreted as the local oxidation of the more reactive remaining W^5+^ states to W^6+^. It has been reported that the W^5+^ states have a stronger interaction with adsorbates in the presence of a high W^5+^ population [46,81].

Humidity is another important parameter that has an impact on the performance of metal oxide gas sensors. It has been reported previously that the electrical conductance of WO_3_ increases in the presence of humidity. The dissociation of water molecules results in the presence of hydroxyl groups OH^-^ and H^+^ that serve as conductive species. At temperatures higher than 200 °C, which is the optimal one for the detection of hydrogen with Nb-WO_3_ devices, there is no more molecular water on the surface of the metal oxide, and hydroxyl groups react as donors [53]. As a result, the presence of water molecules allows a faster restoration of the sensor to its initial state [82].

Table 3 compares the H_2_-sensing performance of pristine and Nb-WO_3_ nanowires with those of some other structures reported previously in [35,36,37,75]. It is evident that the response of proposed Nb-WO_3_ materials are much higher compared to those of pristine and functionalized thin films [35]. Nanostructured materials generally perform better, but only thanks to noble metal functionalization do these devices achieve performances comparable to our Nb-WO_3_. However, most of the investigations found in literature have been performed in dry air environment, which is not realistic in many applications. It is remarkable that WO_3_ + Nb(4) devices exhibit a response of more than 8 × 10^4^ in a humid environment (RH = 50% @ 20 °C). The high surface-to-volume ratio of the nanowire structure further improved by the roughness of the surface, together with the increased depleted regions due to the presence of randomly distributed Nb atoms, allow a more efficient sensing process compared to thin films and nanorods.

## 5. Conclusions

A new generation of high-performing hydrogen gas sensors has been presented, based on the integration of Nb-doped WO_3_ nanowires in conductometric devices by a low-cost thermal oxidation method. HR-TEM and FE-SEM analysis confirm the nanowire-like morphology of the materials, which have diameters of few tens of nanometers and a high surface roughness. The Nb-doping promotes the growth of the nanowires, without affecting the size of the nanostructures. XRD and Raman spectroscopies confirm that the Nb-WO_3_ nanowires have a monoclinic *m*-WO_3_ crystalline structure, but the Nb-doping reduces the overall crystallinity and introduces defects in the structure. However, there is no evidence of secondary phases related to niobium oxides. XPS spectroscopy proves that both Nb and W atoms are fully oxidized in Nb^5+^ and W^6+^ state, respectively. EDX and XPS confirms that the maximum Nb-doping achieved was close to 10%. The presence of Nb-doping further enhances the hydrogen response, reaching the outstanding value of more than 80,000 towards 500 ppm @ 200 °C in the presence of RH = 50% @ 20 °C. Moreover, fabricated devices have proved to be very selective in the presence of interfering compounds such as VOCs, nitrogen dioxide and ammonia. The excellent hydrogen sensing performances of Nb-WO_3_ outmatch similar structures previously reported in literature. This opens the possibility of a mass-scale production of these devices for their integration in commercial equipment, like electronic noses and portable hydrogen sensing devices.

## Figures and Tables

**Table 1:** SEM pictures of sample morphology oxidized at 550 °C under different gas flows for pure WO_3_. Oxidation time was fixed at 1 h. Pressure was set at 100 Pa.

	No Flow	10 sccm O_2_	10 sccm Ar
SEM	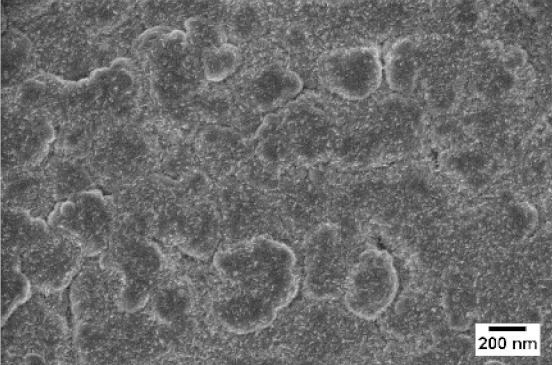	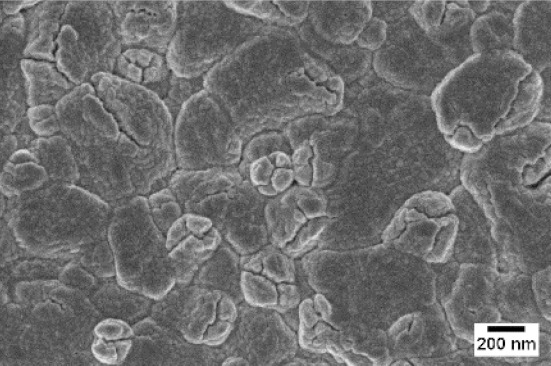	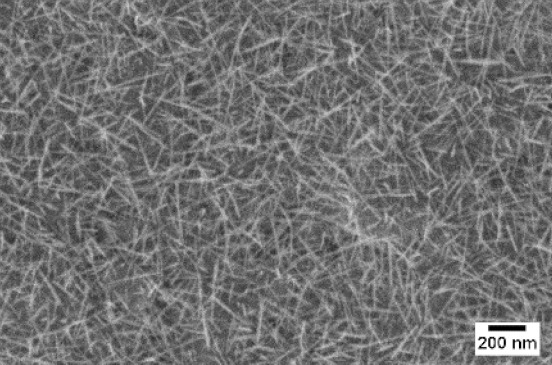

**Table 2:** SEM pictures of sample morphology oxidized at different temperature (500–700 °C) for pristine WO_3_ and Nb-WO_3_. Oxidation time was fixed at 1 h. Gas flow was set at 10 sccm of Argon, resulting in a pressure of 100 Pa.

	WO_3_	WO_3_ + Nb(4)	WO_3_ + Nb(12)
500 °C	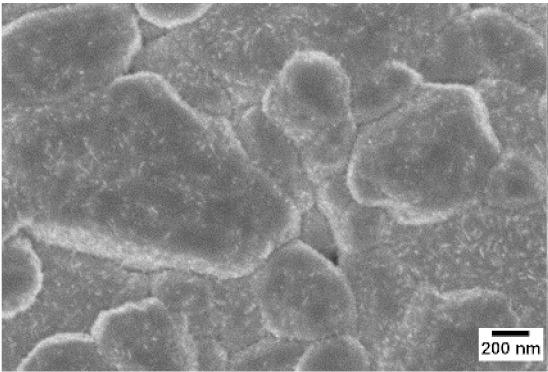	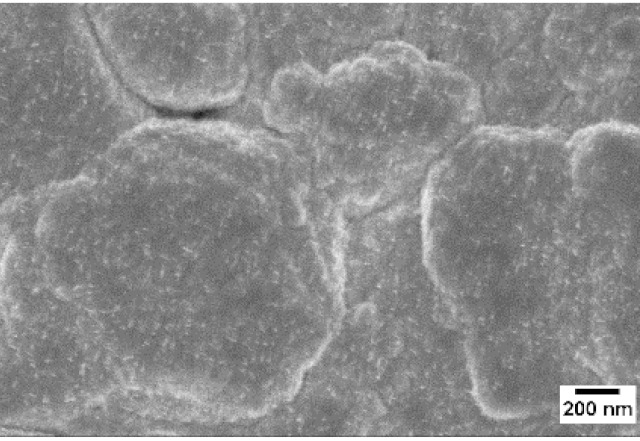	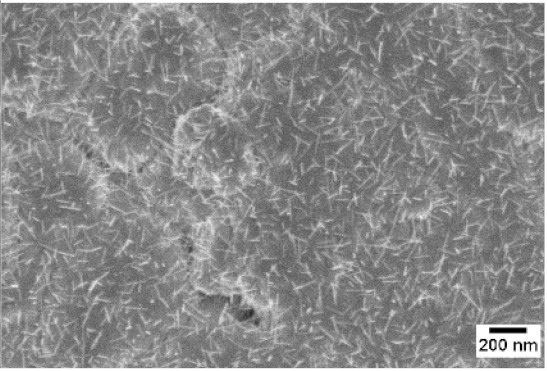
550 °C	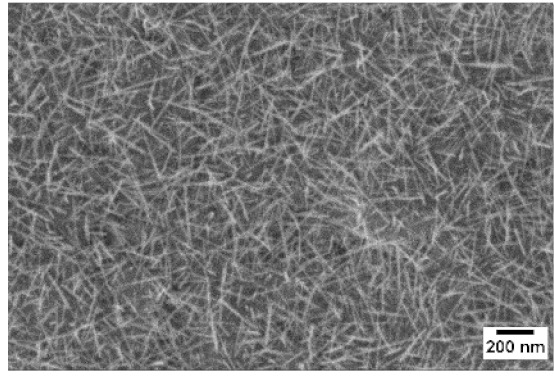	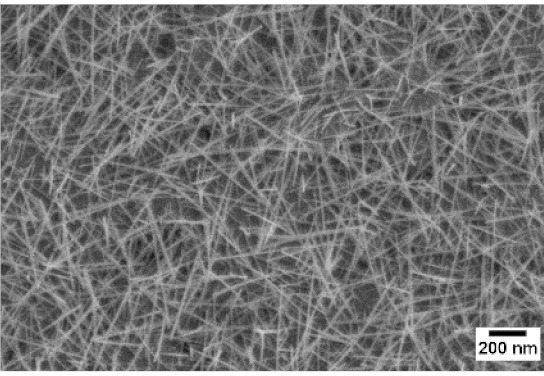	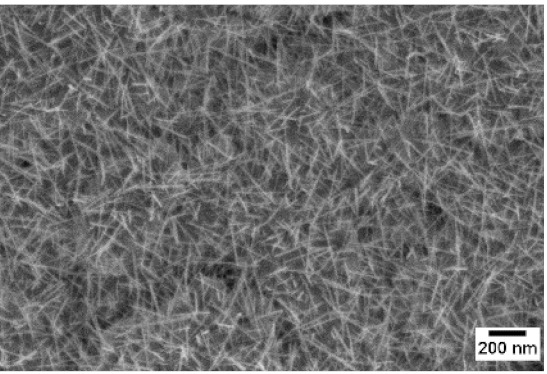
600 °C	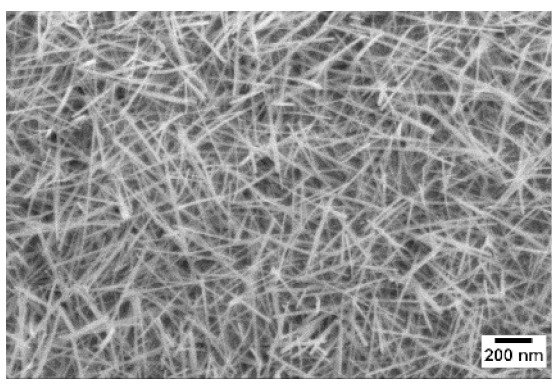	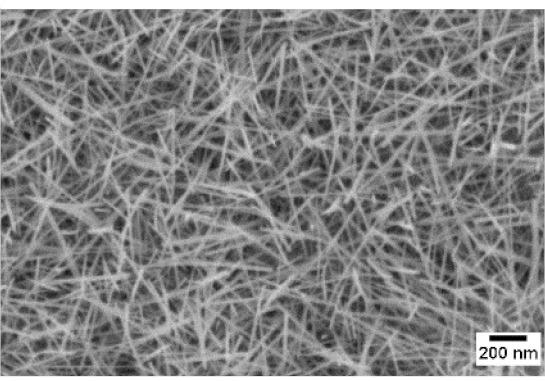	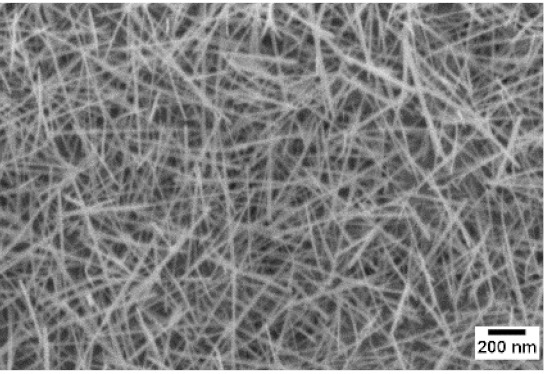
650 °C	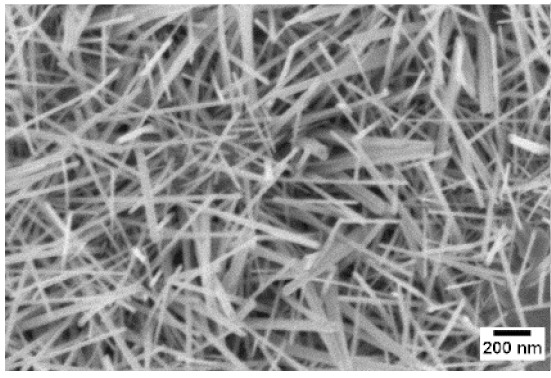	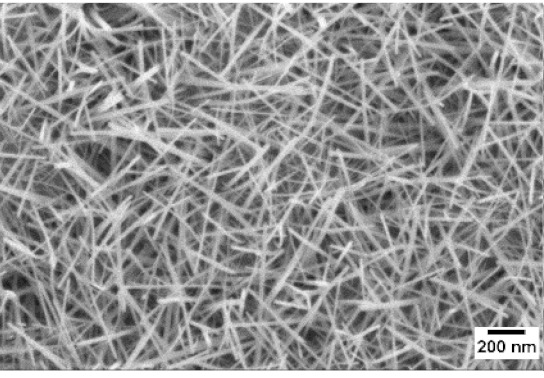	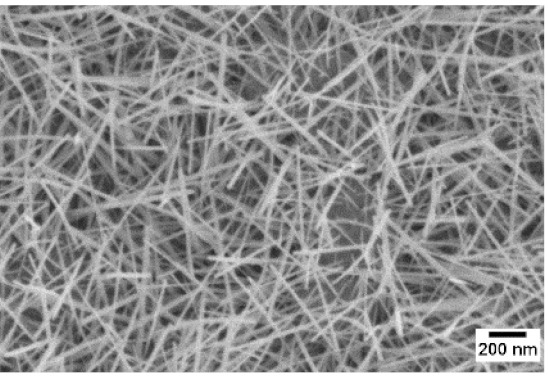
700 °C	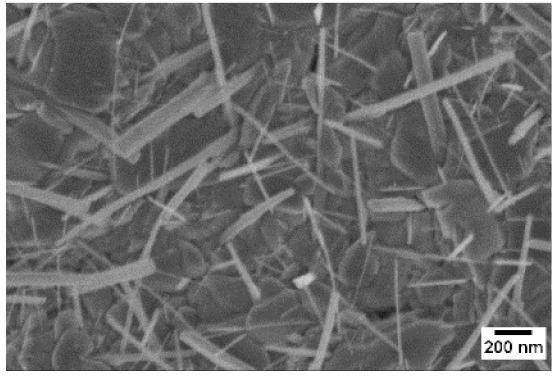	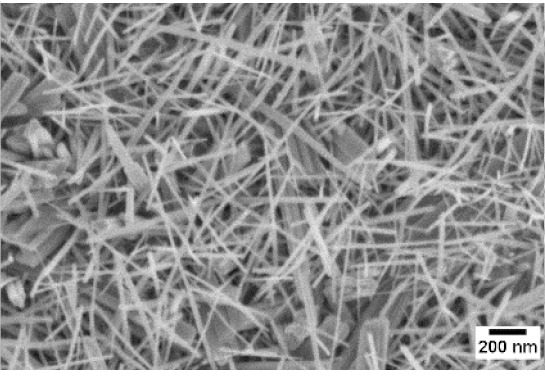	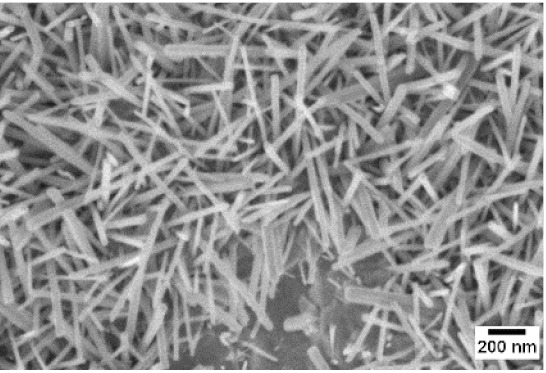

**Table 3:** Response of pristine and WO_3_ + Nb(4) compared to previous literature.

Material	Morphology	H_2_ Gas Concentration	Temperature	Humidity	Response	Ref.
Pd-WO_3_	Ribbon-like	500 ppm	100 °C	Dry air	∆G/G = 80	[36]
Pd-WO_3_/PRGO	Irregular	500 ppm	100 °C	Dry air	∆G/G = 150	[36]
WO_3_	Thin film	0.50%	180 °C	Dry air	∆G/G = 6	[35]
Pt-WO_3_	Thin film	0.50%	70 °C	Dry air	∆G/G = 450	[35]
Au-WO_3_	Thin film	0.50%	262 °C	Dry air	∆G/G = 250	[35]
WO_3_	Nanorods	500 ppm	200 °C	Dry air	R_air_/R_gas_ ≈ 1	[37]
Pt-WO_3_	Nanorods	500 ppm	200 °C	Dry air	R_air_/R_gas_ = 3 × 10^4^	[37]
Pd-WO_3_	Cluster film	2%	80 °C	Dry air	∆G/G = 2.4 × 10^4^	[75]
Pd-WO_3_	Nanoparticles	200 ppm	200 °C	RH = 50%	∆G/G = 20	[38]
Pd-WO3	Nanoneedles	500 ppm	150 °C	RH = 50%	∆G/G = 1670	[31]
WO_3_	Nanowires	500 ppm	200 °C	RH = 50%	∆G/G = 6 × 10^3^	This work
WO_3_ + Nb(4)	Nanowires	500 ppm	200 °C	RH = 50%	∆G/G = 8 × 10^4^	This work

## Supplementary Materials

The following are available online at https://www.mdpi.com/1424-8220/19/10/2332/s1.
