# Supplementary material for: The Influence of Nb on the Synthesis of WO3 Nanowires and the Effects on Hydrogen Sensing Performance
Source: Sensors (Basel). 2019 May 20;19(10):2332. doi: 10.3390/s19102332 (PMC6567310; doi:10.3390/s19102332)
Supplement: Supplementary file 1 [file sensors-19-02332-s001.pdf]

Supplementary Materials

Of

The Influence of Nb on the Synthesis of WO_3 Nanowires and the Effects on Hydrogen Sensing Performance

Dario Zappa [1,*]

[1] SENSOR Laboratory, Department of Information Engineering (DII), University of Brescia, Via Valotti 9, 25133 Brescia, Italy

* Correspondence: dario.zappa@unibs.it; Tel.: +39-030-371-5767

A statistical analysis of the average diameters of the nanowires, at different oxidation temperatures, is reported in Table S1.

Table 1. Statistical distribution of the diameter of the nanowires synthetized at different temperature, for pristine and Nb-WO_3 materials.

**Temperature [°C]**	**WO_3 [nm]**		**WO_3+Nb(4) [nm]**		**WO_3+Nb(12) [nm]**	
	Average	*Stdev*	*Average*	*Stdev*	*Average*	*Stdev*
500					12.6	2.1
550	15.9	3.3	11.6	2.3	13.0	2.6
600	17.2	4.6	19.0	3.2	19.0	4.2
650	29.2	7.0	20.9	4.1	21.5	4.9
700	34.0	21.0	24.7	7.2	35.7	9.4

The coefficients A and B of the estimated power laws for each target compound and material are reported in Table S2.

Table 2. *A* and *B* coefficients of the calibration curves, calculated at 200 °C for pristine and Nb-WO_3 nanowires (RH = 50% @ 20 °C).

Gas Title	Ethanol		Acetone		NO_2		NH_3		H_2	
Sample	A	B	A	B	A	B	A	B	A	B
WO_3	0.049	0.655	<0.001	1.695	1.462	0.818	0.162	1.149	130.990	0.642
WO_3+Nb(4)	0.036	0.696	0.001	1.318	0.742	0.637	0.159	0.997	22.070	1.359
WO_3+Nb(12)	0.024	0.637	0.002	0.958	0.558	0.261	0.063	1.243	2.537	1.415
